# Socio-demographic and environmental determinants of infectious disease morbidity in children under 5 years in Ghana

**DOI:** 10.3402/gha.v8.29349

**Published:** 2015-10-09

**Authors:** Dickson A. Amugsi, Raymond A. Aborigo, Abraham R. Oduro, Victor Asoala, Timothy Awine, Lucas Amenga-Etego

**Affiliations:** 1 African Population and Health Research Centre, Nairobi, Kenya; 2 Navrongo Health Research Centre, Navrongo, Ghana

**Keywords:** morbidity, under 5, children, determinants, Ghana

## Abstract

**Background:**

Globally, diarrhoea and acute respiratory infections (ARIs) have been identified as major threats to child survival. In Ghana, the two conditions are among the top three causes of morbidity and mortality among children under 5 years. An in-depth analysis of the factors associated with these two diseases is warranted, because of their high degree of fatality and also it provides a basis for intervention planning.

**Objectives:**

To investigate socio-demographic and environmental factors associated with infectious disease morbidity in children under 5 years old in Ghana.

**Design:**

Population-based cross-sectional survey. The study sample comprised 2,790 children aged 0–59 months, drawn from the Ghana Demographic and Health Surveys. The mothers reported whether their children under age 5 had been ill with a cough accompanied by short, rapid breathing (ARI), or diarrhoea with the presence of blood or mucus in the stool, in the 2 weeks preceding the survey.

**Results:**

Children in the 6–11, 12–23, and 24–59 months age groups had, respectively, 3.48 (95% CI=2.23, 5.44), 4.57 (95% CI=3.03, 6.90), and 1.93 (95% CI=1.30, 2.87) increased odds of getting diarrhoea infection compared to those in the youngest age category (0–5). Similarly, children in the 6–11, 12–23, and 24–59 months age brackets were, respectively, 2.64 (95% CI=1.76, 3.97), 2.63 (95% CI=1.81, 3.83), and 1.83 (95% CI=1.29, 2.59) times more likely to have cough compared to children in 0–5 months age brackets. Children who were not breastfeeding had higher odds of childhood diarrhoea (OR=1.33, 95% CI=1.03, 1.73) compared to those who were breastfeeding. Compared to children who were living in households without co-wives, children who were living in households with co-wives had 1.74 increased odds of diarrhoea (95% CI=1.33, 2.27). A unit increase in maternal opinion regarding wife beating was associated with 14% reduced odds of diarrhoea (OR=0.86, 95% CI=0.80, 0.91), while a unit change in the women's attitude towards sex index was associated with 14% reduced odds of childhood cough (OR=0.86, 95% CI=0.77, 0.97).

**Conclusions:**

Our results show that breastfeeding, polygamous marriage, and maternal decision-making autonomy are significant predictors of child morbidity. Therefore, implementing effective educational programmes that aim at promoting breastfeeding, empowering women, and discouraging polygamous marriages could help save many children from infectious disease morbidity in Ghana.

Globally, diarrhoea and acute respiratory infections (ARIs) have been identified as major threats to child survival. Recent reports show that diarrhoea, defined as the passage of three or more loose or liquid stools per day, is one of the leading causes of morbidity and mortality in children under 5 years old (1, 2). According to the WHO, an estimated 1.7 billion cases of diarrhoeal disease occur annually worldwide, with approximately 780,000 children dying from the disease (1). The majority of these cases occur in low- and middle-income countries (LMICs). Children under 3 years in LMICs experience about three episodes of diarrhoea annually (1). The situation is more pronounced in the African region where children experience a mean of five episodes (3).

Similarly, ARIs, especially lower respiratory tract infections (LRTIs), are a major cause of death among children under 5 years of age. These conditions are responsible for between 1.9 million and 2.2 million childhood deaths globally (4). It is further documented that 42% of ARI-associated deaths occur in Africa (4).

In Ghana, both diarrhoea and ARIs have been identified as major causes of infant morbidity and mortality. The two conditions are among the top-three causes of morbidity and mortality among children under 5 years (5, 6). An in-depth analysis of the factors associated with these two diseases is warranted, because of their high degree of fatality and also it provides a basis for interventions to tackle them head on.

It is widely recognized that child morbidity in sub-Saharan Africa is influenced by socio-economic, demographic, and environmental factors such as child age, maternal education and occupation, socio-economic status, place of residence, housing conditions, improved toilet facilities and water source, improper disposal of the youngest child's stool, and number of children in the household (7–14).

In Ethiopia, children living in households with improved toilet facilities are less likely to be sick than children in households without any toilet facility (14). This is also the case in Ghana, where children living in houses with toilet facilities are about 50% less likely to contract diarrhoea than their counterparts in homes without toilet facilities (15). Higher morbidity has also been reported in children during complementary feeding as a result of increased exposure to contaminated food. In related studies, low level of maternal education and maternal power to take decisions were observed to be risk factors for childhood morbidity (9, 11). Also, a comparative study conducted in urban areas of Ghana, Egypt, Brazil, and Thailand concluded that children's health is affected by environmental conditions and socio-economic status of households (13).

Although evidence abounds on the influence of socio-demographic and environmental factors on child health, very few studies have investigated these factors using nationally representative data. For example, to the best of our knowledge, this is the first study to have used nationally representative data to simultaneously investigate the factors associated with childhood diarrhoea and cough (a proxy for ARI) in Ghana.

In Ghana, one of the key strategies adopted by the Ministry of Health (MOH) to address morbidity and mortality among children under 5 years of age is the integrated management of childhood illnesses. The strategy aims to reduce childhood deaths, illnesses, and disability through the accurate identification of child morbidities, appropriate combined treatment of these morbidities, counselling of caretakers, and an improved access to care for severely ill children. The strategy also seeks to promote appropriate care-seeking behaviours and improve nutrition and preventative treatment (16). Despite implementing this strategy for over two decades, Ghana would still miss the targets for Millennium Development Goal 4, which aims to reduce by two-thirds the 1990 levels of child mortality by 2015. This calls for deeper insights into both the distant and proximate factors that are associated with these morbidities and efforts towards addressing them.

Identifying the basic environmental and socio-demographic factors that determine childhood morbidity remains important in reducing child mortality. Indeed, several studies have shown that childhood morbidity in LMICs is the result of interactions among behavioural, socio-economic and environmental factors (11, 17, 18). It stands to reason that understanding childhood morbidity involves explaining the relationship and interactions of these factors. This is crucial for more focused implementation of child health interventions for policy formulation and intervention prioritization in Ghana. The main objective of this study was therefore to examine the effect of these factors on diarrhoea and cough using data from the Ghana demographic and health survey (GDHS).

## Materials and methods

### Data sources and study participants

The data for this analysis were drawn from the GDHS, conducted in 2008 as part of the MEASURE DHS international programme. The Ghana DHS employed a two-stage sampling design. The first stage involved selection of clusters from a master sampling frame constructed from the national population and housing census (year 2000 Census). The second stage involved the selection of households from these clusters. All women and men aged 15–49 and 15–59, respectively, in the selected households were eligible to participate in the surveys. The data were collected at two levels – the household and individual levels. At the household level, information was collected on household characteristics such as source of drinking water, toilet facilities, cooking fuel, and assets of the household. At the individual level, questionnaires were administered to eligible women and men to gather information on individual characteristics and health behaviours’, and information on their children. The Ghana DHS collected childhood morbidity data such as diarrhoea and cough. The survey defined diarrhoea as passing of three or more liquid, watery or loose stools per day. Mothers were asked whether any of their children under 5 years of age had diarrhoea during the 2 weeks preceding the survey. If a child had diarrhoea, the mother was asked about the presence of blood or mucus in the stool. Information was also sought on feeding practices during the diarrhoeal episode and about the actions that were taken to treat the diarrhoea. Information on cough was collected by asking mothers whether their children under age 5 years had been ill with a cough accompanied by short, rapid breathing in the 2 weeks preceding the survey. In the 2008 GDHS data, cough was used to estimate ARI (19).

### Study participants

The total sample for this analysis was 2,790 children, aged 0–59 months, who had complete morbidity data and were living with their mothers at the time of the survey. A total of 202 (6.7%) children did not have morbidity data and were therefore excluded in the analysis.

### Ethics statement

Ethical approval was sought from the Ghana Health Service ethical review committee (GHS-ERC) before the survey was conducted. Written informed consent was also obtained from study participants before they were allowed to participate in the study. The data were completely anonymous and therefore the authors did not seek further ethical clearance before the use of the data.

### Outcome and explanatory variables

The main outcome variables for this analysis were diarrhoea and cough. The mother's ‘yes’ response to the child suffering from any of the two conditions was coded ‘1’ while a ‘no’ response was coded ‘0’. The explanatory variables used in the analysis included child level factors (age, sex, and vaccination status), maternal level factors (education, occupation, literacy, women's role in household decision-making index, opinion regarding wife beating index, and justified to refuse sexual intercourse with husband index), and household level factors (disposal of the youngest child's stool, type of toilet facility, source of drinking water, floor material, presence of co-wives, household wealth, and place of residence). Some of the variables were recoded. The recoding was done to make the results more interpretable and to preserve sample size for the analysis. Type of toilet facilities and source of drinking water were recoded into ‘improved’ and ‘unimproved’, respectively (20), while the disposal of the youngest child's stool was recoded into ‘appropriate’ and ‘inappropriate’ disposal. Additionally, floor material was recoded into ‘dirt’ and non-dirt. Three decision-making autonomy indices (women's role in household decision-making, the number of reasons that justify wife beating in the respondent's opinion; and the respondent's opinion on the number of circumstances under which a wife is justified in refusing to have sexual intercourse with her husband) were created based on empowerment variables captured in the data set, and according to the DHS recommended techniques (19). The details on the construction of these indexes can be found elsewhere (21).

### Statistical analysis

SPSS version 22 was used in analysing the data. The analysis involved a number of stages. The first stage was a descriptive analysis to examine the characteristics of the sample and to estimate the prevalence of diarrhoea and cough by selected socio-demographic and environmental factors. The second stage was to conduct bivariate analysis of the associations between socio-demographic and environmental factors, and the outcome variables. Only significant factors (*p*<0.05) or those that were considered critical (e.g. biological factors) were used in the multivariate analysis. We used logistic regression to conduct the multivariate analysis because the outcome variable is dichotomous. A total of eight regression models, four per each morbidity outcome, were constructed. The first model in each case contains biological factors of the child (age and sex) and the mother (age). In the second model, child vaccination (polio 3 and DPT 3) and breastfeeding status variables were introduced. Maternal level factors (education, occupation, literacy, women's role in household decision-making, opinion regarding wife beating, and justified to refuse sexual intercourse with husband) were introduced in the third model, adjusting for factors in the first and second models. In the final and fourth model, household level factors (disposal of the youngest child's stool, type of toilet facility, source of drinking water, floor material, presence of co-wives, household wealth, and place of residence) were included in the analysis, accounting for factors in the first, second and third models. Association was considered statistically significant at *p*<0.05, and the estimated coefficients (β) when exponentiated were interpreted as the odds of diarrhoeal and cough morbidity relative to morbidity in the reference group. All analyses were adjusted for design effects (sample weight, strata, and cluster). This was conducted using SPSS Complex Samples package. We first created a Complex Samples ‘plan file’, which was then used to run the multivariate analysis. Multicollinearity was investigated but none observed.

## Results

### Descriptive analysis of the sample

The average age of the children used in this analysis was 28 months, with 51% being males (Table 1). Seventy-two percent and 75% of the children in this population were given polio 3 and DPT/Hep B/Influenza 3 vaccinations, respectively, while 91% received BCG at birth. Forty-six percent and 23% did not have access to improved sanitation and drinking water source, respectively. Also, 49% of the mothers did not dispose of the youngest child's stool appropriately.

**Table 1:** Descriptive analysis of the sample (*n*=2,790): continuous and categorical variables

Variables	Mean±SD/%
Child age (in months)	27.74±17.58
Mother age (in years)	30.08±7.01
Child sex	
Male	51.0
Female	49.0
Vaccinations	
Polio 3 (yes)	71.8
DPT/Hep B/influenza 3 (yes)	75.2
BCG (yes)	91.3
Maternal education	
No education	37.8
Primary	24.1
Secondary +	38.0
Disposal of the youngest child's stool	
Inappropriate	49.0
Type of toilet facility	
Unimproved	46.2
Source of drinking water	
Unimproved	22.5
Place of residence	
Urban	33.4
Rural	66.6

Mean±SD=means and standard deviations of continuous variables.

### Prevalence of diarrhoea and cough (ARI)

The overall prevalence for diarrhoea and cough among children under 5 years of age in the sample was 21 and 22%, respectively (Table 2). Diarrhoea prevalence peaked (32%) between the ages of 12 and 23 months, and declined significantly (17%) between the ages of 24 and 59 months (Table 2). This was also the case for cough. There was no sex difference in diarrhoea prevalence. Expectedly, children of mothers without formal education were more prone to diarrhoea than those whose mothers had some education (*p*=0.001). The reverse was the case with cough – children of mothers with formal education had higher prevalence of cough than those without education. Children of mothers who disposed of the youngest child's stool inappropriately had higher prevalence of diarrhoea than those who used appropriate disposal methods (24% versus 17%, *p*=0.001). This was reflected in the use of unimproved sanitation facilities, where prevalence was higher among children in households with unimproved sanitation facilities than those in households with improved facilities (23% versus 19%, *p*=0.005).

**Table 2:** Prevalence of diarrhoea and cough by selected environmental and socio-demographic factors (*n*=2,790)

	Diarrhoea		Cough	
		
Variables	%	*p*	%	*p*
Child age (in months)				
0–5	9.5	0.001	13.0	0.001
6–11	26.7		28.0	
12–23	32.4		27.9	
24 +	17.2		20.9	
Sex of child				
Male	21.0	0.90	22.5	0.87
Female	20.8		22.3	
Maternal age (in years)				
15–24	21.3	0.611	25.9	0.018
25–34	20.1		20.3	
35–49	21.8		23.1	
Education level				
No education	24.4	0.001	20.2	0.008
Primary	20.6		26.6	
Secondary +	17.6		21.9	
Disposal of the youngest child's stool				
Appropriate	17.3	0.001	22.4	0.97
Inappropriate	24.3		22.4	
Type of toilet facility				
Improved	18.9	0.005	23.4	0.19
Unimproved	23.2		21.3	
Source of drinking water				
Improved	21.0	0.95	23.1	0.13
Unimproved	20.9		20.3	
Floor material				
Non-dirt	20.2	0.076	22.8	0.51
Dirt	23.4		21.6	
Presence of co-wives				
There are no co-wives	18.1	0.001	21.7	0.58
There are co-wives	27.4		23.0	
Type of place of residence				
Urban	18.5	0.033	24.8	0.034
Rural	22.0		21.2	
Overall prevalence	20.9		22.4	

### Multivariate logistic regression analysis of determinants of diarrhoea

Table 3 presents the results of the multivariate logistic regression analysis. The results in the first model showed that child age was significantly associated with diarrhoea morbidity. Children in the 6–11, 12–23, and 24–59 age groups had, respectively, 3.48 (95% CI=2.23, 5.44), 4.57 (95% CI=3.03, 6.90), and 1.93 (95% CI=1.30, 2.87) increased odds of getting diarrhoea infection compared to those in the youngest age category (0–5 months). These statistical significant associations remained in models 2, 3, and 4. In model 2, children who did not receive polio 3 were 32% less likely to suffer from diarrhoea (OR=0.68, 95% CI=0.46, 0.99) compared to those who were vaccinated. In the same model, children who did not receive complete DPT/Hep B/Influenza vaccines were 1.5 times more likely to experience diarrhoea (95% CI=1.03, 2.26) compared to children who received the full vaccination. These significant associations disappeared when maternal level factors were introduced in the model (model 3). Non-breastfed children were more at risk of diarrhoea than breastfed children (OR=1.33, 95% CI=1.03, 1.73). This association remained in model 4. In the third model, maternal decision-making autonomy regarding wife beating was significantly associated with decreased risk of childhood diarrhoea. A unit increase in maternal opinion regarding wife beating was associated with 14% reduced odds of diarrhoea (OR=0.86, 95% CI=0.80, 0.91). This association remained significant in the final model (model 4). In model 4, compared to children who were living in households without co-wives, children who were living in households with co-wives had 1.74 increased odds of diarrhoea (95% CI=1.33, 2.27).

**Table 3:** Multiple logistic regression analysis of the socio-demographic and environmental determinants of childhood diarrhoea (*n*=2,790)

	Model 1		Model 2		Model 3		Model 4	
				
Variables	OR	95% CI for OR	OR	95% CI for OR	OR	95% CI for OR	OR	95% CI for OR
*Maternal and child biological factors*								
Child age (in months)								
0–5	Ref	Ref	Ref	Ref	Ref	Ref	Ref	Ref
6–11	3.48	2.23, 5.44**	3.54	2.20, 5.71**	3.49	2.05, 5.96**	3.31	1.87, 5.85**
12–23	4.57	3.03, 6.90**	4.70	3.00, 7.36**	4.92	2.97, 8.16**	4.55	2.64, 7.78**
24 +	1.93	1.30, 2.87**	2.00	1.31, 3.07**	2.05	1.27, 3.31**	1.96	1.18, 3.25**
Sex of child								
Male	Ref	Ref	Ref	Ref	Ref	Ref	Ref	Ref
Female	0.94	0.78, 1.14	0.94	0.77, 1.13	0.99	0.80, 1.22	0.98	0.78, 1.23
Maternal age in years								
15–24	Ref	Ref	Ref	Ref	Ref	Ref	Ref	Ref
25–34	0.97	0.76, 1.23	0.98	0.76, 1.25	1.04	0.78, 1.37	1.14	0.83, 1.57
35–49	1.18	0.90, 1.55	1.18	0.90, 1.54	1.22	0.90, 1.66	1.19	0.84, 1.69
*Child level factors*								
Breastfeeding status	–	–						
Breastfeeding	–	–	Ref	Ref	Ref	Ref	Ref	Ref
Not breastfeeding	–	–	1.18	0.93, 1.50	1.33	1.03, 1.73**	1.48	1.11, 1.98**
Polio 3 vaccination	–	–						
Vaccinated	–	–	Ref	Ref	Ref	Ref	Ref	Ref
Not vaccinated	–	–	0.68	0.46, 0.99**	0.72	0.48, 1.08	0.77	0.50, 1.19
DPT/Hep B/influenza 3 vaccination	–	–						
Vaccinated	–	–	Ref	Ref	Ref	Ref	Ref	Ref
Not vaccinated	–	–	1.53	1.03, 2.26**	1.41	0.92, 2.16	1.28	0.81, 2.01
*Maternal level factors*								
Education level	–	–	–	–				
Secondary +	–	–	–	–	Ref	Ref	Ref	Ref
Primary	–	–	–	–	0.93	0.67, 1.29	0.91	0.63, 1.31
No education	–	–	–	–	1.11	0.81, 1.52	1.03	0.71, 1.49
Has a say in decision-making index	–	–	–	–	0.94	0.87, 1.01	0.99	0.91, 1.09
Wife beating not justified index	–	–	–	–	0.86	0.80, 0.91**	0.86	0.80, 0.92**
Can refuse husband sex index	–	–	–	–	0.89	0.79, 1.01	0.93	0.82, 1.07
*Household level factors*								
Disposal of the youngest child's stool	–	–	–	–	–	–		
Appropriate	–	–	–	–	–	–	Ref	Ref
Inappropriate	–	–	–	–	–	–	1.24	0.98, 1.58
Type of toilet facility	–	–	–	–	–	–		
Improved	–	–	–	–	–	–	Ref	Ref
Unimproved	–	–	–	–	–	–	0.98	0.74, 1.30
Source of drinking water	–	–	–	–	–	–		
Improved	–	–	–	–	–	–	Ref	Ref
Unimproved	–	–	–	–	–	–	0.97	0.74, 1.28
Floor material	–	–	–	–	–	–		
Non-dirt	–	–	–	–	–	–	Ref	Ref
Dirt	–	–	–	–	–	–	1.05	0.77, 1.42
Presence of co-wives	–	–	–	–	–	–		
There are no co-wives	–	–	–	–	–	–	Ref	Ref
There are co-wives	–	–	–	–	–	–	1.74	1.33, 2.27**
Household wealth index	–	–	–	–	–	–		
Richest	–	–	–	–	–	–	Ref	Ref
Rich	–	–	–	–	–	–	1.22	0.72, 2.05
Middle	–	–	–	–	–	–	1.77	1.03, 3.06**
Poor	–	–	–	–	–	–	1.45	0.80, 2.65
Poorest	–	–	–	–	–	–	1.72	0.88, 3.35
Type of place of residence	–	–	–	–	–	–		
Urban	–	–	–	–	–	–	Ref	Ref
Rural	–	–	–	–	–	–	0.97	0.68, 1.37

OR, odds ratios; CI, confidence intervals.

** Significant at *p*<0.05.

### Multivariate logistic regression analysis of determinants of cough (ARI)

Table 4 presents the results of the multivariate logistic regression analysis. The analysis in model 1 showed that child age is significantly associated with child cough (ARI). Children in the 6–11, 12–23, and 24–59 months age brackets were, respectively, 2.64 (95% CI=1.76, 3.97), 2.63 (95% CI=1.81, 3.83), and 1.83 (95% CI=1.29, 2.59) times more likely to have cough compared to children in the 0–5 months age brackets. Children who were not breastfeeding had higher odds of contracting cough than those who were breastfeeding. This association remained in model 3, but disappeared in model 4. Compared to children of mothers aged 15–24 years, children of mothers aged 25–34 years were 27% less likely to suffer from cough (OR=0.73, 95% CI=0.58, 0.92). This association remained after adjusting for child level factors (vaccination and breastfeeding status), maternal level factors, and household level factors in models 2, 3, and 4, respectively. Opinion on wife beating and attitude towards sex indexes were associated with reduced odds of cough in children. A unit increase in the wife-beating index was associated with 12% reduced odds of childhood cough (OR=0.88, 95% CI=0.83, 0.94), while a unit change in the women's attitude towards sex index was associated with 14% reduced odds of childhood cough (OR=0.86, 95% CI=0.77, 0.97). These statistical significant associations remained in the final model. In model 4, compared to children who lived in the richest households, children who lived in poor and poorest households were almost two times more likely to suffer from childhood cough.

**Table 4:** Multiple logistic regression analysis of the socio-demographic and environmental determinants of childhood cough (*n*=2,790)

	Model 1		Model 2		Model 3		Model 4	
				
Variables	OR	95% CI for OR	OR	95% CI for OR	OR	95% CI for OR	OR	95% CI for OR
*Maternal and child biological factors*								
Child age (in months)								
0–5	Ref	Ref	Ref	Ref	Ref	Ref	Ref	Ref
6–11	2.64	1.76, 3.97**	2.64	1.70, 4.09**	2.92	1.81, 4.71**	2.63	1.59, 4.33**
12–23	2.63	1.81, 3.83**	2.62	1.74, 3.96**	2.73	1.74, 4.30**	2.46	1.53, 3.96**
24 +	1.83	1.29, 2.59**	1.85	1.26, 2.70**	1.92	1.27, 2.92**	1.76	1.14, 2.73**
Sex of child								
Male	Ref	Ref	Ref	Ref	Ref	Ref	Ref	Ref
Female	0.98	0.89, 1.18	0.98	0.81, 1.17	1.02	0.84, 1.25	0.98	0.80, 1.22
Maternal age in years								
15–24	Ref	Ref	Ref	Ref	Ref	Ref	Ref	Ref
25–34	0.73	0.58, 0.92**	0.74	0.59, 0.93**	0.76	0.58, 0.98**	0.69	0.52, 0.92**
35–49	0.88	0.68, 1.13	0.87	0.67, 1.12	0.98	0.74, 1.32	0.89	0.65, 1.21
*Child level factors*								
Breastfeeding status	–	–						
Breastfeeding	–	–	Ref	Ref	Ref	Ref	Ref	Ref
Not breastfeeding	–	–	1.28	1.02, 1.60**	1.31	1.02, 1.68**	1.24	0.95, 1.62
Polio 3 vaccination	–							
Vaccinated	–	–	Ref	Ref	Ref	Ref	Ref	Ref
Not vaccinated	–	–	087	0.61, 1.24	0.81	0.55, 1.19	0.81	0.54, 1.21
DPT/HepB/influenza 3 vaccination	–	–						
Vaccinated	–	–	Ref	Ref	Ref	Ref	Ref	Ref
Not vaccinated	–	–	1.15	0.79, 1.67	1.27	0.85, 1.90	1.30	0.85, 1.99
*Maternal level factors*								
Education level	–	–	–	–				
Secondary +	–	–	–	–	Ref	Ref	Ref	Ref
Primary	–	–	–	–	1.40	1.03, 1.89**	1.53	1.10, 2.14**
No education	–	–	–	–	0.99	0.73, 1.37	1.08	0.75, 1.55
Has a say in decision-making index	–	–	–	–	0.98	0.92, 1.06	1.02	0.93, 1.11
Wife beating not justified index	–	–	–	–	0.88	0.83, 0.94**	0.89	0.83, 0.96**
Can refuse husband sex index	–	–	–	–	0.86	0.77, 0.97**	0.86	0.76, 0.97**
*Household level factors*								
Disposal of the youngest child's stool	–	–	–	–	–	–		
Appropriate	–	–	–	–	–	–	Ref	Ref
Inappropriate	–	–	–	–	–	–	1.02	0.81, 1.28
Type of toilet facility	–	–	–	–	–	–		
Improved	–	–	–	–	–	–	Ref	Ref
Unimproved	–	–	–	–	–	–	0.80	0.61, 1.05
Source of drinking water	–	–	–	–	–	–		
Improved	–	–	–	–	–	–	Ref	Ref
Unimproved	–	–	–	–	–	–	0.90	0.69, 1.17
Floor material	–	–	–	–	–	–		
Non-dirt	–	–	–	–	–	–	Ref	Ref
Dirt	–	–	–	–	–	–	0.86	0.64, 1.17
Presence of co-wives	–	–	–	–	–	–		
There are no co-wives	–	–	–	–	–	–	Ref	Ref
There are co-wives	–	–	–	–	–	–	1.13	0.56, 1.48
Household wealth index	–	–	–	–	–	–		
Richest	–	–	–	–	–	–	Ref	Ref
Rich	–	–	–	–	–	–	1.52	0.94, 2.24
Middle	–	–	–	–	–	–	1.54	0.94, 2.38
Poor	–	–	–	–	–	–	1.81	1.08, 3.04**
Poorest	–	–	–	–	–	–	1.98	1.09, 3.60**
Type of place of residence	–	–	–	–	–	–		
Urban	–	–	–	–	–	–	Ref	Ref
Rural	–	–	–	–	–	–	0.77	0.57, 1.06

OR, odds ratios; CI, confidence intervals.

** Significant at *p*<0.05.

## Discussion

This study investigated the influence of socio-demographic and environmental factors on childhood morbidity in Ghana. The results show that the risk of child morbidity in the 2-week reference period peaks at 12–23 months, with children between the ages of 24 and 59 months having the lowest risk of diarrhoea and cough. The high risk of morbidity in the 12–23 months age brackets could be due to loss of innate immunity and/or exposure to different types of infections from eating contaminated food prepared with unclean water and in unhealthy environment as reported in other studies (10, 22–24). On the other hand, low risk of morbidity among older children could be due to the immunity the children build over time, which enables their bodies to fight off infectious agents from the environment. Our results are similar to the findings of previous studies in sub-Saharan Africa. A study in Eritrea found that the risk of diarrhoea infection peaks at age 6–11 months (10). This was also the case in Nigeria, where prevalence of diarrhoea was found to be high among children aged 6–11 months old (25). In Ethiopia, the morbidity peak occurred among children 6–11 and 12–23 months, respectively (26). The preceding literature together with the findings of the current study illuminate the crucial role age plays in child health outcomes. These findings suggest that interventions to address child morbidity in Ghana could take into account the age of the child.

Similarly, breastfeeding status of the child shows a significant association with morbidity. Children who were still breastfeeding were less likely to have diarrhoea compared to those who were not breastfeeding. This protective effect was achieved after maternal and household level factors were included in the models. The opposite happened in the case of cough, where the introduction of household level factors in the analysis (model 4) eliminated the estimated protective effect of breastfeeding. This suggests that the protective effect of breastfeeding in the second and third models could be attributed to other variables. It is important to note that although breastfeeding is not significant in the initial model for diarrhoea and the final model for cough, it is appropriate to conclude that breastfeeding has a protective effect on child morbidity, but that the effect is dependent to some extent on what variables are adjusted for in the analysis. The protective effect of breastfeeding on child morbidity has been widely documented in both high-income (27, 28) and LMICs (29). A study conducted in the United Kingdom indicated varying degrees of protection across levels of breastfeeding exposure (27). This study estimated that 53% of diarrhoea morbidity could be prevented each month by exclusive breastfeeding and 31% by partial breastfeeding. The authors also observed that 27% of LRTIs could be prevented each month by exclusive breastfeeding and 25% by partial breastfeeding (27). Similar results were reported in two systematic review studies which found a large body of evidence for the protective effects of breastfeeding against infectious disease morbidity in young children (30, 31). Thus, public health interventions that focus on increasing exclusive breastfeeding and encouraging mothers to continue breastfeeding are more likely to reduce infection-related childhood morbidity.

Our analysis also showed significant associations between maternal decision-making ability and childhood morbidity. Mothers who have improved scores on decision-making autonomy indexes (opinion on sex with husband and opinion on wife beating) have children who are less likely to experience childhood diarrhoea and cough. A study in rural India observed that low level of maternal power to take decisions is associated with increased risk of child morbidity (9). Similarly, studies have documented relationships between maternal autonomy and utilization of maternal and child healthcare services (32–35), which are critical for the health and well-being of the child. One plausible explanation why women autonomy is important for child health is the observation by UNICEF (36) that women's ability to influence decision-making in the household determines how resources are allocated for caring practices such as feeding, prenatal and birthing care, curative and preventive health-seeking behaviour for children. Furthermore, women's ability to control resources in the household has a positive effect on their own health and well-being, which in turn impacts positively on their children's health (36). Indeed, the negative effect of women's autonomy on childcare, including utilization of child healthcare services, which are important for good child health outcomes has long been established (37). In a predominantly patriarchal society like Ghana, health decisions and control of resources are largely in the hands of men. Women depend on their counterparts for resources to care for the family and to seek health care (38–40). Also, polygamy is common in rural Ghana and women in polygamous marriages are less autonomous compared to those in monogamous marriages (41). Therefore, efforts towards implementing intervention programmes that are geared towards empowering women should be encouraged.

The number of co-wives in the household is also an important determinant of childhood morbidity. Children who live in polygamous households are more likely to contract diarrhoeal infection compared to those living in monogamous households. These findings are consistent with some studies in the literature. For instance, a study in Ghana revealed that children in polygamous households were at higher risk of mortality than those in monogamous households (42). The possible reasons for these differences are many and varied. Some authors posit that polygamy limits women's access to financial resources from their spouses and also forces them to share their limited resources in the household and thus exacerbate their impoverishment (43). This could potentially affect women's ability to give proper care to their children, thereby resulting in poor health (36). Polygamy can also create overcrowding in the household, which presents conducive environmental conditions for the transmission of infectious agents (44).

The household wealth index (an indicator of socio-economic status) is also another important determinant of child morbidity. Our analysis found a graded relationship between household wealth index and childhood cough (e.g. children in the poorest wealth quintile are at higher risk than those in the poor quintile and poor quintile worse than the middle quintile and so on). The risk of infectious disease illness among those in the poorest wealth quintile may be attributable to greater exposure to infectious agents (44). This is because the poor are likely to live in poor environmental conditions such as poor sanitation, and engage in poor hygienic practices, which might increase their exposure to infectious agents. Additionally, it is documented that poor families often have more children and live in more crowded houses; both environmental conditions are conducive for the transmission of infectious agents (44, 45). Also, poor people may not be able to afford nutritious food and this can lead to poor nutritional status, and inadequate nutrition is known to suppress the immune system's ability to fight off infections (44, 46). The graded relationships between household wealth and child morbidity have been reported elsewhere. Margolis et al. (47) observed that the incidence of lower respiratory illness was 1.41 in the low socio-economic group, 1.26 in the middle group, and 0.67 in the high group.

This study has both strengths and limitations. The study used data that are representative at the national, regional, and rural-urban levels. This implies that the findings of our study can be generalized to all children under 5 years old in Ghana. Another important strength is that even though data used for the study are not clinical or longitudinal data, they lend a hand for assessing the determinants of childhood morbidity in the 2 weeks prior to the survey. Such findings may be of relevance for health policy planning and intervention in Ghana.

One important limitation associated with this study is that the data used for the study are from a cross-sectional survey; therefore, it is difficult to account for seasonal variations in the occurrence of child morbidity. A longitudinal study may be more suitable to provide data covering different seasons. Also, during the DHS survey, the children were not clinically examined. The morbidity was measured based on mothers’ report of their children's health in the past 2 weeks preceding the survey. These questions measure mother's perception of her child's health instead of morbidity according to a clinical examination. As people from different backgrounds are likely to have different perception of childhood illness, the mother's report of child ill health may not be the same across different socio-economic groups. These differences could result in either under-reporting or over-reporting of childhood illnesses, and consequently affect the prevalence reported in this paper. Hence, the prevalence estimates reported here should be interpreted with caution. Another important limitation is recall bias. Mothers could have forgotten the morbidity event during the interview leading to misreporting on the occurrence of illness. We, however, believe that, as have been generally the case in reporting such events, 2 weeks was recent enough for most mothers to have reported appropriately. It is our view that most of these shortcomings could be addressed in a well-designed longitudinal study. For now, cross-sectional data such as the Ghana DHS offers a platform to investigate factors that determine childhood morbidity in Ghana and the estimates obtained may be relevant for health intervention programmes in Ghana.

## Conclusions and recommendations

### Conclusions

Child age is an important predictor of both diarrhoea and ARI morbidity in children. Non-breastfeeding and polygamous marriage place children at high risk of childhood diarrhoea. Mothers who have high decision-making autonomy have children who are less likely to experience diarrhoea and cough infections, while the risk of childhood cough is associated with children in the lower wealth quintiles.

### Recommendations

The findings of this study have important policy implications for health programme design and intervention planning:

- These findings suggest that age is an important factor to consider for implementing interventions to address child morbidity in Ghana.
- Implementing effective educational programmes that aim at promoting and prolonging breastfeeding may have a considerable effect on child survival in Ghana.
- Efforts should be made to implement intervention programmes that are geared towards empowering women to be able to actively participate in household decision-making, as well as stand for themselves. One of the key ways to empower women is the promotion of girl child education.
- Effective educational programmes that discourage polygamy through a focused behaviour change communication having this straight-to-the-point message: one man one wife – having one wife is good for your child's health.
- A longitudinal study may be the best design to provide data in different seasons by including behavioural and other relevant factors.
